# Gender Differences in Elderly With Subjective Cognitive Decline

**DOI:** 10.3389/fnagi.2018.00166

**Published:** 2018-06-04

**Authors:** Lijun Wang, Ting Tian, Alzheimer’s Disease Neuroimaging Initiative

**Affiliations:** ^1^Institutes of Brain Science, Fudan University, Shanghai, China; ^2^Department of Neurology, Zhongshan Hospital, Fudan University, Shanghai, China; ^3^State Key Laboratory of Medical Neurobiology, Fudan University, Shanghai, China; ^4^Department of Neurology, The Second Hospital, Lanzhou University, Lanzhou, China

**Keywords:** gender, significant memory concern, cognitive function, hippocampus, entorhinal cortex, medial temporal lobe

## Abstract

**Objective**: Subjective cognitive decline (SCD), also known as significant memory concern (SMC), has been suggested as a manifestation of Alzheimer’s Disease (AD) preceding mild cognitive impairment (MCI). This study assessed the impact of gender on cognition, amyloid accumulation, the volumes of hippocampus, entorhinal cortex (EC), fusiform and medial temporal lobe (MTA) and cerebrospinal fluid (CSF) pathology biomarkers in patients reporting SMC.

**Methods**: Twenty-nine males (mean age ± SD: 72.3 ± 5.7 years) and 40 females (mean age ± SD: 71.0 ± 5.1 years) with SMC from the AD Neuroimaging Initiative (ADNI) were included in the study. We explored the gender discrepancies in cognition, [^18^F] AV45 amyloid positivity, volumes of hippocampus, EC, fusiform and MTA and CSF biomarkers.

**Results**: Compared with females, males showed significantly worse performance in Assessment Scale-cognitive subscale 13 (ADAS-13; *P* = 0.004) and lower amyloid deposition (*P* < 0.001). However, females showed greater advantage on the task of Rey Auditory Verbal Learning Test-5 (RAVLT-5) sum (*P* = 0.021), RAVLT-immediate recall (*P* = 0.010) and reduced volumes of the hippocampus, EC, fusiform and MTA (*P* = 0.001, *P* < 0.001, *P* < 0.001, *P* = 0.007) than males. No gender differences were found in CSF Aβ42, CSF Tau and CSF P-tau (*P* = 0.264, *P* = 0.454, *P* = 0.353).

**Conclusions**: These findings highlight that gender discrepancies should be considered in the interpretation of cognitive measures when evaluating SMC.

## Introduction

Significant memory concern (SMC; also known as subjective cognitive decline (SCD) or subjective memory impairment), is defined as a self-reported cognitive complaints in the absence of objective cognitive deficits, which is common in older adults (Jessen et al., 2014; Jenkins et al., 2015). Recent mounting evidences indicated that SMC is a risk factor for future accelerated cognitive decline and progression to preclinical or clinical state of Alzheimer’s disease (AD), with AD-type changes in amyloid deposition, neuroimaging and cerebrospinal fluid (CSF) biomarkers (Petersen, 2000; Visser et al., 2009; Reisberg et al., 2010; Perrotin et al., 2012; Scheef et al., 2012; Wang et al., 2012; Mitchell et al., 2014). Taken together, these results suggested that SMC might be an initial symptomatic indicator of preclinical AD (Jessen et al., 2014).

Gender-specific discrepancies in mild cognitive impairment (MCI) and AD have been observed (Roberts et al., 2012; Lin et al., 2015). Thus, sex-specific research in SMC is crucial to ensure early correct detection and pre-clinical intervention. However, to date, few studies have focused on the role of gender in SMC across a comprehensive profile of the cognitive assessment, neuroimaging and CSF AD biomarkers.

Therefore, the purpose of the current study was to go further to analyze whether the gender discrepancies are related to neuropsychological performance, CSF and positron emission tomography (PET) and magnetic resonance imaging (MRI) biomarkers of AD pathology in older adults reporting SMC.

## Materials and Methods

### ADNI Study Design

Data used in the preparation of this article were obtained from the AD Neuroimaging Initiative (ADNI) database^1^ during January 2018. The data collectors were blind to participant information during the experiments. The ADNI was launched in 2003 as a public-private partnership, led by Principal Investigator Michael W. Weiner, MD. The primary goal of ADNI has been to test whether serial MRI, PET, other biological markers and clinical and neuropsychological assessment can be combined to measure the progression of MCI and early AD. The Principal Investigator of this initiative is Michael W. Weiner, MD, VA Medical Center and University of California-San Francisco. ADNI is a global research effort that actively supports the investigation and development of treatments that slow or stop the progression of AD and subjects have been recruited from over 50 sites across the US and Canada. The overall goal of ADNI is to determine biomarkers for use in AD clinical treatment trials. To date, it has three phases: ADNI1, ADNI GO and ADNI2, consisting of cognitively normal (CN) individuals, early MCI (EMCI), to late MCI (LMCI), and dementia or AD. For more information, see www.adni-info.org. This study was carried out in accordance with the recommendations of each ADNI site. The protocol was approved by the ADNI. All subjects gave written informed consent in accordance with the Declaration of Helsinki.

### Subjects

The current study sample consisted of 69 ADNI-2 participants, including 29 males and 40 females. Participants were selected if they were diagnosed as having SMC. Diagnosis was made using the standard criteria described in the ADNI-2 procedures manual^2^. Briefly, SMC participants had subjective memory concerns as evaluated using the Cognitive Change Index (CCI; total score from first 12 items >16), which was based on selected items from a larger compilation of measures analyzed in an independent sample (Saykin et al., 2006), but no informant-reported memory complaints, and normal cognitive performance on the Wechsler Logical Memory Delayed Recall (LM-Delayed) and the Mini-Mental State Exam (MMSE).

### Neuropsychological Assessment

All participants underwent a standardized cognitive evaluation including the following items: (1) Global cognitive function: MMSE (Folstein et al., 1975), Montreal Cognitive Assessment (MoCA; Nasreddine et al., 2005), AD Assessment Scale-cognitive subscale 13 (ADAS-13; Mohs et al., 1997), Global Clinical Dementia Rating Scale (CDR-SB; Morris, 1993); (2) Memory: the Rey Auditory Verbal Learning Test (RAVLT), including trials 1–5 total recall (RAVLT-5 sum), 5-min delayed recall (RAVLT-immediate recall), 30-min delayed recall (RAVLT-delayed recall), yes-no recognition (RAVLT-recognition; Schmidt, 1996); (3) Attention/executive function: the Trail Making Test-A and B (TMT-A/B; Reitan, 1955); (4) Language: animal fluency, 30-item Boston Naming Task (BNT-30; Domoto-Reilly et al., 2012); (5) Visuospatial: clock Drawing Test (CDT; Brodaty and Moore, 1997); (6) 15-item Geriatric Depression Scale (GDS-15; Yesavage et al., 1982), Functional Assessment Questionnaire (FAQ; Pfeffer et al., 1982), Neuropsychiatric Inventory (NPI; Cummings et al., 1994).

### Apolipoprotein E Genotyping

Apolipoprotein E (APOE; gene map locus 19q13.2) genotypes of the study subjects were obtained from the ADNI database^1^. All subjects were classified as APOE ε4 carriers with phenotypes ε2/ε4, ε3/ε4, and ε4/ε4, APOE ε4 non-carriers group with ε2/ε2, ε2/ε3 and ε3/ε3 genotypes.

### Detection of CSF Aβ42, Tau and P-tau

The CSF Aβ42, Tau and P-tau immunoassays were used following a Roche Study Protocol at the University of Pennsylvania/ADNI Biomarker Laboratory, according to the preliminary kit manufacturer’s instructions, as described in previous studies (Bittner et al., 2016). Values are given in pg/mL for both tau and Aβ42.

### [^18^F] AV 45 (Florbetapir) PET Scans

[^18^F] AV 45 (Florbetapir) PET data were processed and acquired as described previously (Landau et al., 2012, 2013b). Mean florbetapir standard uptake value ratios (SUVRs) were measured within four regions (frontal, anterior cingulate, precuneus, and parietal cortex) and normalized to the whole cerebellum reference region. Participants were classified as amyloid positivity when the SUVRs were >1.11, and amyloid negativity when the SUVRs were ≤1.11 based on a previously established threshold (Landau et al., 2013a). For more detailed information about PET protocols and data, see the ADNI website^3^.

### Quantification of Volumes of Hippocampus, Entorhinal Cortex, Fusiform and Medial Temporal Lobe

The ADNI neuroimaging standardized procedure has been described in great detail elsewhere (Jack et al., 2008). ADNI-2 MRI data were acquired on a 3 Tesla MRI scanners using T1-weighted sagittal 3D magnetization-prepared rapid gradient-echo (MPRAGE) sequences. Cortical reconstruction and volumetric segmentation were obtained using FreeSurfer version 5.1 image analysis suite in ADNI 2^4^ (McDonald et al., 2009), as described in previous reports (Fischl et al., 2001, 2002; Fleisher et al., 2005; Han et al., 2006). In this study, hippocampus, EC, fusiform and medial temporal lobe (MTA) volumes were evaluated. Further details on ADNI imaging protocols can be found at http://adni.loni.usc.edu/methods/documents/mri-protocols/.

### Statistical Analysis

Demographic and clinical variables were compared between genders in the overall sample using Student’s *t*-test by mean ± standard deviation (SD) according to the distribution, Mann–Whitney test for skewed distribution variables by median (M) and interquartile range (IQR), Chi-square test for categorical variables. All statistics were performed using SPSS software (version 23.0; IBM SPSS). All calculated tests were two-sided and statistical significance was set at *P* < 0.05. Figures were produced using GraphPad Prism 6.

## Results

### Demographic Characteristics by Gender

The overall sample was comprised of 69 participants including 29 males and 40 females were downloaded from the ADNI website. Socio-demographics and clinical characteristics of the study sample are presented in Table 1. In brief, females were less educated compared to males (*P* = 0.036) and males were reported with more alcohol drinking (*P* = 0.038). No gender differences were found in other variables (all *P* > 0.05).

**Table 1:** Demographic characteristics of subjects included in the study.

Characteristics	Total	Males (*n* = 29)	Females (*n* = 40)	*P* value
Age, years	71.6 ± 5.3	72.3 ± 5.7	71.0 ± 5.1	0.318
Education, years	16 (16–18)	18 (16–20)	16 (15–18)	0.036
Race, *n* (% White)	64 (92.8)	26 (40.6)	38 (59.4)	0.708
Ethnicity, *n* (% Not Hisp/Latino)	66 (95.7)	29 (43.9)	37 (56.1)	0.363
Marital status, *n* (% Married)	50 (72.5)	23 (46)	27 (54)	0.278
Right handedness, *n* (%)	58 (84.1)	26 (44.8)	32 (55.2)	0.280
APOE ε4 carriers, *n* (%)	25 (36.2)	8 (32)	17 (68)	0.203
Smoking, *n* (%)	30 (43.5)	15 (50)	15 (50)	0.239
Alcohol abuse, *n* (%)	3 (4.3)	3 (100)	0 (0)	0.038
Hypertension, *n* (%)	35 (50.7)	15 (42.9)	20 (57.1)	0.888
SBP (mmHg)	135.4 ± 17.5	132.1 ± 17.6	137.7 ± 17.2	0.191
DBP (mmHg)	73.3 ± 8.7	72.9 ± 9.4	73.5 ± 8.3	0.792

*Abbreviations: APOE, apolipoprotein E; DBP, diastolic blood pressure; SBP, systolic blood pressure. Data are described as mean ± SD, median (M) and the interquartile range (IQR) unless otherwise specified. P values tested by Student’s t-test, Mann-Whitney test and Chi-square test*.

### Cognitive Profiles by Gender

**Figure 1:**
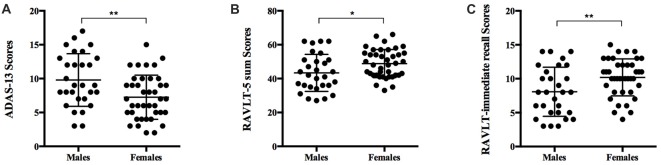
Comparison of neuropsychological measures in men and women with significant memory concern (SMC). Scatter plots displaying cognitive function in males and females. **(A)** Men had significantly worse cognitive function as measured by ADAS-13 (*P* = 0.004). **(B)** Women showed greater advantages on RAVLT-5 sum (*P* = 0.021). **(C)** Women excelled at RAVLT-immediate recall (*P* = 0.010). ADAS-13, Alzheimer’s Disease Assessment Scale-cognitive subscale 13; RAVLT, Rey Auditory Verbal Learning Test. *P* values tested by Student’s *t*-test. *P < 0.05, **P < 0.01.

Gender differences in neuropsychological performances in the study sample are demonstrated in Table 2 and Figure 1. Men performed worse on ADAS-13 (*P* = 0.004), while women had statistically significant better cognitive function on RAVLT-5 sum (*P* = 0.021) and RAVLT-immediate recall (*P* = 0.010). However, there were no gender differences in performance on MMSE, MoCA, CDR-SB, RAVLT-delayed recall, RAVLT-recognition, TMT-A, TMT-B, Animals fluency, BNT-30, CDT, GDS-15, FAQ, NPI (*P* = 0.917, *P* = 0.098, *P* = 0.408, *P* = 0.053, *P* = 0.064, *P* = 0.229, *P* = 0.888, *P* = 0.685, *P* = 0.920, *P* = 0.350, *P* = 0.554, *P* = 0.843, *P* = 0.632).

**Table 2:** Clinical assessments by gender.

Variables	Total	Males (*n* = 29)	Females (*n* = 40)	*P* value
MMSE	29 (29–30)	29 (28–30)	29 (29–30)	0.917
MoCA	25.9 ± 2.4	25.4 ± 2.5	26.4 ± 2.3	0.098
ADAS-13	8.3 ± 3.7	9.8 ± 3.9	7.3 ± 3.3	0.004
CDR-SB	0 (0–0)	0 (0–0)	0 (0–0)	0.408
RAVLT-5 sum	46.6 ± 9.8	43.4 ± 10.9	48.9 ± 8.3	0.021
RAVLT-immediate recall	9.3 ± 3.3	8.1 ± 3.6	10.2 ± 2.7	0.010
RAVLT-delayed recall	7.4 ± 4.0	6.2 ± 4.4	8.2 ± 3.4	0.053
RAVLT-recognition	14 (12–14.5)	13 (10.5–14)	14 (12.25–15)	0.064
TMT-A	32.3 ± 10.6	34.1 ± 12.6	31.0 ± 8.9	0.229
TMT-B	82.2 ± 39.3	81.4 ± 32.0	82.8 ± 44.3	0.888
Animals fluency	20.9 ± 5.0	21.2 ± 4.9	20.7 ± 5.1	0.685
BNT-30	29 (28–30)	29 (28–29.5)	29 (28–30)	0.920
CDT	5 (4–5)	5 (4–5)	5 (5–5)	0.350
GDS-15	1 (0–2)	1 (0.5–1.5)	1 (0–2)	0.554
FAQ	0 (0–0)	0 (0–0.5)	0 (0–0)	0.843
NPI	0 (0–2.5)	0 (0–3)	0 (0–1.75)	0.632

*Abbreviations: ADAS-13, Alzheimer’s Disease Assessment Scale-cognitive subscale 13; BNT-30, Boston Naming Task; CDR-SB, Global Clinical Dementia Rating Scale; CDT, Clock Drawing Test; FAQ, Functional Assessment Questionnaire; GDS-15, Geriatric Depression Scale; MMSE, Mini-Mental State Examination; MoCA, Montreal Cognitive Assessment; NPI, Neuropsychiatric Inventory; RAVLT, Rey Auditory Verbal Learning Test; TMT, The Trail Making Test. Data are presented as mean ± SD, median (M) and the interquartile range (IQR). P values tested by Student’s t-test, Mann-Whitney test*.

### CSF Biomarkers

There were no significant differences in CSF Aβ42, Tau and P-tau levels between males and females (*P* = 0.264, *P* = 0.454, *P* = 0.353; see Table 3).

**Table 3:** Measurements of cerebrospinal fluid (CSF) biomarkers, positron emission tomography (PET) and magnetic resonance imaging (MRI) in the subjects.

Variables	Total	Males (*n* = 29)	Females (*n* = 40)	*P* value
CSF Aβ42 (pg/mL)	1370.6 ± 614.2	1468.2 ± 587.2	1299.9 ± 630.9	0.264
CSF Tau (pg/mL)	243.3 ± 94.3	233.3 ± 90.6	250.6 ± 97.4	0.454
CSF P-tau (pg/mL)	22.4 ± 10.1	21.0 ± 9.1	23.3 ± 10.8	0.353
[^18^F] AV45 SUVRs	1.06 (1.01–1.21)	1.01 (0.98–1.09)	1.11 (1.03–1.36)	<0.001
Aβ positivity, *n* (%)	23 (33.3)	4 (17.4)	19 (82.6)	0.003
Hippocampus (mm^3^)	7678.7 ± 911.7	8095.7 ± 950.5	7376.4 ± 760.1	0.001
Entorhinal (mm^3^)	3905.1 ± 568.2	4215.7 ± 532.8	3679.9 ± 485.1	<0.001
Fusiform (mm^3^)	18853.0 ± 2209.3	20071.6 ± 2185.2	17969.5 ± 1782.7	<0.001
MTA (mm^3^)	20823.3 ± 2776.2	21863.6 ± 3072.6	20069.1 ± 2296.1	0.007

*Abbreviations: MTA, medial temporal atrophy; SUVR, standardized uptake values ratio. *P* values tested by Student’s *t*-test, Mann-Whitney test and Chi-square test*.

### Aβ Deposition in Different Groups

Significant decreases in [^18^F] AV45 SUVRs were found in males compared with females (*P* < 0.001). Aβ positivity was detected in 17.4% of males, 82.6% of females respectively. The percentage of Aβ positive subjects was significantly higher in females vs. males (*P* = 0.003; Table 3).

### Volumes of Hippocampus, Entorhinal Cortex, Fusiform and Medial Temporal Lobe by Gender

The volumes of hippocampus, EC, fusiform, medial temporal lobe (MTA) are illustrated in Table 3. There were significantly larger volumes of hippocampus, EC, fusiform and MTA in men compared to women (*P* = 0.001, *P* < 0.001, *P* < 0.001, *P* = 0.007).

## Discussion

In the present study, we explored a statistically significant gender discrepancy in cognition function, Aβ deposition and brain volume in older adults with SMC. The results confirmed that women with SMC outperformed men with SMC on the tasks of RAVLT-5 sum and RAVLT-immediate recall, while the advantage was eliminated on the task of RAVLT-delayed recall and a floor effect might limit interpretation. However, we found that men with SMC were associated with worse performance on ADAS-13. No significant differences were observed between males and females on other cognitive domains. Our results are in concordance with previous studies showing that the female advantage in verbal memory task was more apparent than men (Herlitz et al., 1997; Sundermann et al., 2016a,b). These observations suggest that gender discrepancies among SMC subjects might be appropriate to a specific cognitive domain. If so, then implementing sex-adjusted norms in clinical memory tests might ameliorate the diagnostic accuracy in women.

Females with SMC participants showed increased Aβ deposition relative to males. Elevated risk of AD was in women compared to men, although the underlying mechanism remains elusive as previously reported (Seshadri et al., 2006). However, we did not discover differences in CSF Aβ42, Tau and P-tau between males and females with subjective cognitive impairment. It is noteworthy that we observed significant reduction in volumetric measurements of hippocampus, EC, fusiform gyrus and MTA in females with SMC subjects. Consistent with the cognitive reserve theory (Klonoff and Landrine, 1992; Stern et al., 1994, 2003; Stern, 2002), better premorbid performance of women on verbal episodic memory tests might confer an advantage in the ability to maintain normal verbal memory performance despite reduced volumes of hippocampal, EC, fusiform gyrus, MTA and accumulating AD pathology (Sundermann et al., 2016a,b, 2017). However, women may have more accelerated decline once neuropathology reached a threshold level (Klonoff and Landrine, 1992; Stern et al., 1994).

Several limitations should be mentioned in the current study. First, a simple cross-sectional design used in the study does not definitively permit the theory that female advantage in verbal memory may act as a specific form of cognitive reserve, further longitudinal researches would be more necessary to closely confirm the conclusions. Second, The ADNI cohort included a potential selection bias based on the fact that ADNI participants had a high education level (Petersen et al., 2010; Grill et al., 2013), which may affect the generalizability to a greater population sections. Third, the relatively small group size could limit the interpretation of our results, larger sample of numbers need to be collected in future studies.

## Conclusion

In summary, the present results highlighted the urgent need to consider the sex differences in cognition evaluation, which contributes to clinical diagnosis even in preclinical stages, such as SMC.

## Author Contributions

LW: conceived and designed the studies; wrote the article; is the corresponding author. LW and TT: performed the research; analyzed the data. All authors approved the final version of the manuscript for publication.

## Conflict of Interest Statement

The authors declare that the research was conducted in the absence of any commercial or financial relationships that could be construed as a potential conflict of interest.

## Footnotes

^1^adni.loni.usc.edu

^2^http://www.adni-info.org

^3^http://adni.loni.usc.edu/methods/

^4^http://surfer.nmr.mgh.harvard.edu/

## References

[B1] Bittner T. Zetterberg H. Teunissen C. E. Ostlund R. E. Jr. Militello M. Andreasson U.. (2016). Technical performance of a novel, fully automated electrochemiluminescence immunoassay for the quantitation of β-amyloid (1–42) in human cerebrospinal fluid. Alzheimers Dement. 12, 517–526. 10.1016/j.jalz.2015.09.009 26555316

[B2] Brodaty H. Moore C. M. (1997). The clock drawing test for dementia of the Alzheimer’s type: a comparison of three scoring methods in a memory disorders clinic. Int. J. Geriatr. Psychiatry 12, 619–627. 10.1002/(sici)1099-1166(199706)12:6<619::aid-gps554>3.3.co;2-8 9215942

[B3] Cummings J. L. Mega M. Gray K. Rosenberg-Thompson S. Carusi D. A. Gornbein J. (1994). The neuropsychiatric inventory: comprehensive assessment of psychopathology in dementia. Neurology 44, 2308–2314. 10.1212/wnl.44.12.2308 7991117

[B4] Domoto-Reilly K. Sapolsky D. Brickhouse M. Dickerson B. C. Alzheimer’s Disease Neuroimaging Initiative. (2012). Naming impairment in Alzheimer’s disease is associated with left anterior temporal lobe atrophy. Neuroimage 63, 348–355. 10.1016/j.neuroimage.2012.06.018 22728617 PMC3665400

[B5] Fischl B. Liu A. Dale A. M. (2001). Automated manifold surgery: constructing geometrically accurate and topologically correct models of the human cerebral cortex. IEEE Trans. Med. Imaging 20, 70–80. 10.1109/42.906426 11293693

[B6] Fischl B. Salat D. H. Busa E. Albert M. Dieterich M. Haselgrove C.. (2002). Whole brain segmentation: automated labeling of neuroanatomical structures in the human brain. Neuron 33, 341–355. 10.1016/S0896-6273(02)00569-X 11832223

[B7] Fleisher A. Grundman M. Jack C. R. Jr. Petersen R. C. Taylor C. Kim H. T.. (2005). Sex, apolipoprotein E epsilon 4 status and hippocampal volume in mild cognitive impairment. Arch. Neurol. 62, 953–957. 10.1001/archneur.62.6.953 15956166

[B8] Folstein M. F. Folstein S. E. McHugh P. R. (1975). “Mini-mental state”. A practical method for grading the cognitive state of patients for the clinician. J. Psychiatr. Res. 12, 189–198. 10.1016/0022-3956(75)90026-6 1202204

[B9] Grill J. D. Di L. Lu P. H. Lee C. Ringman J. Apostolova L. G.. (2013). Estimating sample sizes for predementia Alzheimer’s trials based on the Alzheimer’s disease neuroimaging initiative. Neurobiol. Aging 34, 62–72. 10.1016/j.neurobiolaging.2012.03.006 22503160 PMC3412892

[B10] Han X. Jovicich J. Salat D. van der Kouwe A. Quinn B. Czanner S.. (2006). Reliability of MRI-derived measurements of human cerebral cortical thickness: the effects of field strength, scanner upgrade and manufacturer. Neuroimage 32, 180–194. 10.1016/j.neuroimage.2006.02.051 16651008

[B11] Herlitz A. Nilsson L. G. Bäckman L. (1997). Gender differences in episodic memory. Mem. Cognit. 25, 801–811. 10.3758/bf03211324 9421566

[B12] Jack C. R. Jr. Bernstein M. A. Fox N. C. Thompson P. Alexander G. Harvey D.. (2008). The Alzheimer’s disease neuroimaging initiative (ADNI): MRI methods. J. Magn. Reson. Imaging 27, 685–691. 10.1002/jmri.21049 18302232 PMC2544629

[B13] Jenkins A. Bayer A. Tree J. Tales A. (2015). Self-reported memory complaints: implications from a longitudinal cohort with autopsies. Neurology 84:2384. 10.1212/wnl.0000000000001677 26054896

[B14] Jessen F. Wolfsgruber S. Wiese B. Bickel H. Mosch E. Kaduszkiewicz H.. (2014). AD dementia risk in late MCI, in early MCI, and in subjective memory impairment. Alzheimers Dement. 10, 76–83. 10.1016/j.jalz.2012.09.017 23375567

[B15] Klonoff E. A. Landrine H. (1992). Sex roles, occupational roles, and symptom-reporting: a test of competing hypotheses on sex differences. J. Behav. Med. 15, 355–364. 10.1007/bf00844728 1404351

[B16] Landau S. M. Breault C. Joshi A. D. Pontecorvo M. Mathis C. A. Jagust W. J.. (2013a). Amyloid-β imaging with Pittsburgh compound B and florbetapir: comparing radiotracers and quantification methods. J. Nucl. Med. 54, 70–77. 10.2967/jnumed.112.109009 23166389 PMC3747730

[B17] Landau S. M. Lu M. Joshi A. D. Pontecorvo M. Mintun M. A. Trojanowski J. Q.. (2013b). Comparing positron emission tomography imaging and cerebrospinal fluid measurements of β-amyloid. Ann. Neurol. 74, 826–836. 10.1002/ana.23908 23536396 PMC3748164

[B18] Landau S. M. Mintun M. A. Joshi A. D. Koeppe R. A. Petersen R. C. Aisen P. S.. (2012). Amyloid deposition, hypometabolism, and longitudinal cognitive decline. Ann. Neurol. 72, 578–586. 10.1002/ana.23650 23109153 PMC3786871

[B19] Lin K. A. Choudhury K. R. Rathakrishnan B. G. Marks D. M. Petrella J. R. Doraiswamy P. M. (2015). Marked gender differences in progression of mild cognitive impairment over 8 years. Alzheimers Dement. 1, 103–110. 10.1016/j.trci.2015.07.001 26451386 PMC4593067

[B20] McDonald C. R. McEvoy L. K. Gharapetian L. Fennema-Notestine C. Hagler D. J. Jr. Holland D.. (2009). Regional rates of neocortical atrophy from normal aging to early Alzheimer disease. Neurology 73, 457–465. 10.1212/WNL.0b013e3181b16431 19667321 PMC2727145

[B21] Mitchell A. J. Beaumont H. Ferguson D. Yadegarfar M. Stubbs B. (2014). Risk of dementia and mild cognitive impairment in older people with subjective memory complaints: meta-analysis. Acta Psychiatr. Scand. 130, 439–451. 10.1111/acps.12336 25219393

[B22] Mohs R. C. Knopman D. Petersen R. C. Ferris S. H. Ernesto C. Grundman M.. (1997). Development of cognitive instruments for use in clinical trials of antidementia drugs: additions to the Alzheimer’s Disease Assessment Scale that broaden its scope. The Alzheimer’s Disease Cooperative Study. Alzheimer Dis. Assoc. Disord. 11, S13–S21. 10.1097/00002093-199700112-00003 9236948

[B23] Morris J. C. (1993). The clinical dementia rating (CDR): current version and scoring rules. Neurology 43, 2412–2414. 10.1212/WNL.43.11.2412-a 8232972

[B24] Nasreddine Z. S. Phillips N. A. Bedirian V. Charbonneau S. Whitehead V. Collin I.. (2005). The Montreal Cognitive Assessment, MoCA: a brief screening tool for mild cognitive impairment. J. Am. Geriatr. Soc. 53, 695–699. 10.1111/j.1532-5415.2005.53221.x 15817019

[B25] Perrotin A. Mormino E. C. Madison C. M. Hayenga A. O. Jagust W. J. (2012). Subjective cognition and amyloid deposition imaging: a Pittsburgh Compound B positron emission tomography study in normal elderly individuals. Arch. Neurol. 69, 223–229. 10.1001/archneurol.2011.666 22332189 PMC4004919

[B26] Petersen R. C. (2000). Mild cognitive impairment: transition between aging and Alzheimer’s disease. Neurologia 15, 93–101. 10.1002/0470846453.ch14 10846869

[B27] Petersen R. C. Aisen P. S. Beckett L. A. Donohue M. C. Gamst A. C. Harvey D. J.. (2010). Alzheimer’s disease neuroimaging initiative (ADNI): clinical characterization. Neurology 74, 201–209. 10.1212/WNL.0b013e3181cb3e25 20042704 PMC2809036

[B28] Pfeffer R. I. Kurosaki T. T. Harrah C. H. Jr. Chance J. M. Filos S. (1982). Measurement of functional activities in older adults in the community. J. Gerontol. 37, 323–329. 10.1093/geronj/37.3.323 7069156

[B29] Reisberg B. Shulman M. B. Torossian C. Leng L. Zhu W. (2010). Outcome over seven years of healthy adults with and without subjective cognitive impairment. Alzheimers Dement. 6, 11–24. 10.1016/j.jalz.2009.10.002 20129317 PMC3873197

[B30] Reitan R. M. (1955). The relation of the trail making test to organic brain damage. J. Consult. Psychol. 19, 393–394. 10.1037/h0044509 13263471

[B31] Roberts R. O. Geda Y. E. Knopman D. S. Cha R. H. Pankratz V. S. Boeve B. F.. (2012). The incidence of MCI differs by subtype and is higher in men: the Mayo Clinic Study of Aging. Neurology 78, 342–351. 10.1212/WNL.0b013e3182452862 22282647 PMC3280046

[B32] Saykin A. J. Wishart H. A. Rabin L. A. Santulli R. B. Flashman L. A. West J. D.. (2006). Older adults with cognitive complaints show brain atrophy similar to that of amnestic MCI. Neurology 67, 834–842. 10.1212/01.wnl.0000234032.77541.a2 16966547 PMC3488276

[B33] Scheef L. Spottke A. Daerr M. Joe A. Striepens N. Kölsch H.. (2012). Glucose metabolism, gray matter structure, and memory decline in subjective memory impairment. Neurology 79, 1332–1339. 10.1212/wnl.0b013e31826c1a8d 22914828

[B34] Schmidt M. (1996). Rey Auditory Verbal Learning Test: A Handbook. Los Angeles, CA: Psychological Services.

[B35] Seshadri S. Beiser A. Kelly-Hayes M. Kase C. S. Au R. Kannel W. B.. (2006). The lifetime risk of stroke: estimates from the Framingham Study. Stroke 37, 345–350. 10.1161/01.STR.0000199613.38911.b2 16397184

[B36] Stern Y. (2002). What is cognitive reserve? Theory and research application of the reserve concept. J. Int. Neuropsychol. Soc. 8, 448–460. 10.1017/s1355617702813248 11939702

[B37] Stern Y. Gurland B. Tatemichi T. K. Tang M. X. Wilder D. Mayeux R. (1994). Influence of education and occupation on the incidence of Alzheimer’s disease. JAMA 271, 1004–1010. 10.1001/jama.271.13.1004 8139057

[B38] Stern Y. Zarahn E. Hilton H. J. Flynn J. DeLaPaz R. Rakitin B. (2003). Exploring the neural basis of cognitive reserve. J. Clin. Exp. Neuropsychol. 25, 691–701. 10.1076/jcen.25.5.691.14573 12815506

[B39] Sundermann E. E. Biegon A. Rubin L. H. Lipton R. B. Landau S. Maki P. M. (2017). Does the female advantage in verbal memory contribute to underestimating Alzheimer’s disease pathology in women versus men? J. Alzheimers Dis. 56, 947–957. 10.3233/JAD-160716 28106548 PMC7644197

[B40] Sundermann E. E. Biegon A. Rubin L. H. Lipton R. B. Mowrey W. Landau S.. (2016a). Better verbal memory in women than men in MCI despite similar levels of hippocampal atrophy. Neurology 86, 1368–1376. 10.1212/WNL.0000000000002570 26984945 PMC4831033

[B41] Sundermann E. E. Maki P. M. Rubin L. H. Lipton R. B. Landau S. Biegon A. (2016b). Female advantage in verbal memory: evidence of sex-specific cognitive reserve. Neurology 87, 1916–1924. 10.1212/WNL.0000000000003288 27708128 PMC5100712

[B42] Visser P. J. Verhey F. Knol D. L. Scheltens P. Wahlund L. O. Freund-Levi Y.. (2009). Prevalence and prognostic value of CSF markers of Alzheimer’s disease pathology in patients with subjective cognitive impairment or mild cognitive impairment in the DESCRIPA study: a prospective cohort study. Lancet Neurol. 8, 619–627. 10.1016/S1474-4422(09)70139-5 19523877

[B43] Wang Y. West J. D. Flashman L. A. Wishart H. A. Santulli R. B. Rabin L. A.. (2012). Selective changes in white matter integrity in MCI and older adults with cognitive complaints. Biochim. Biophys. Acta 1822, 423–430. 10.1016/j.bbadis.2011.08.002 21867750 PMC3235544

[B44] Yesavage J. A. Brink T. L. Rose T. L. Lum O. Huang V. Adey M.. (1982). Development and validation of a geriatric depression screening scale: a preliminary report. J. Psychiatr. Res. 17, 37–49. 10.1016/0022-3956(82)90033-4 7183759

